# Feature Selection Methods for Early Predictive Biomarker Discovery Using Untargeted Metabolomic Data

**DOI:** 10.3389/fmolb.2016.00030

**Published:** 2016-07-08

**Authors:** Dhouha Grissa, Mélanie Pétéra, Marion Brandolini, Amedeo Napoli, Blandine Comte, Estelle Pujos-Guillot

**Affiliations:** ^1^INRA, UMR1019, UNH-MAPPINGClermont-Ferrand, France; ^2^INRA, UMR1019, Plateforme d'Exploration du MétabolismeClermont-Ferrand, France; ^3^LORIAVandoeuvre-lès-Nancy, France

**Keywords:** feature selection, metabolomics, biomarker discovery, prediction, formal concept analysis, machine learning, univariate statistics, visualization

## Abstract

Untargeted metabolomics is a powerful phenotyping tool for better understanding biological mechanisms involved in human pathology development and identifying early predictive biomarkers. This approach, based on multiple analytical platforms, such as mass spectrometry (MS), chemometrics and bioinformatics, generates massive and complex data that need appropriate analyses to extract the biologically meaningful information. Despite various tools available, it is still a challenge to handle such large and noisy datasets with limited number of individuals without risking overfitting. Moreover, when the objective is focused on the identification of early predictive markers of clinical outcome, few years before occurrence, it becomes essential to use the appropriate algorithms and workflow to be able to discover subtle effects among this large amount of data. In this context, this work consists in studying a workflow describing the general feature selection process, using knowledge discovery and data mining methodologies to propose advanced solutions for predictive biomarker discovery. The strategy was focused on evaluating a combination of numeric-symbolic approaches for feature selection with the objective of obtaining the best combination of metabolites producing an effective and accurate predictive model. Relying first on numerical approaches, and especially on machine learning methods (SVM-RFE, RF, RF-RFE) and on univariate statistical analyses (ANOVA), a comparative study was performed on an original metabolomic dataset and reduced subsets. As resampling method, LOOCV was applied to minimize the risk of overfitting. The best k-features obtained with different scores of importance from the combination of these different approaches were compared and allowed determining the variable stabilities using Formal Concept Analysis. The results revealed the interest of RF-Gini combined with ANOVA for feature selection as these two complementary methods allowed selecting the 48 best candidates for prediction. Using linear logistic regression on this reduced dataset enabled us to obtain the best performances in terms of prediction accuracy and number of false positive with a model including 5 top variables. Therefore, these results highlighted the interest of feature selection methods and the importance of working on reduced datasets for the identification of predictive biomarkers issued from untargeted metabolomics data.

## Introduction

Metabolomics is a powerful phenotyping tool in nutrition research to better understand the biological mechanisms involved in the pathophysiological processes and identify biomarkers of metabolic deviations (Ramautar et al., 2013). It can be described as a global analysis of small molecules present in a biofluid (blood, urine, saliva…), which are produced or modified as a result of stimuli (nutritional intervention, drug, genetic perturbations…) (Nicholson et al., 1999; Fiehn et al., 2000). Among different approaches, the untargeted strategy is a data driven approach dedicated to biomarker discovery. Based on the use of multiple analytical platforms, such as mass spectrometry (MS), it allows detecting thousands of features and offers the possibility of characterizing global alterations associated with disease conditions, and of identifying early and/or predictive biomarkers of disease development (Mamas et al., 2011). Such platforms generate massive and complex data that need analyses and integration to extract the biologically meaningful information. Metabolomic data processing is probably one of the most challenging step of this approach, because of some intrinsic characteristics: data are generated from an instrumental signal, noisy, and contain a high number of highly correlated variables compared to the number of individuals. Despite a lot of chemometrics tools available in the literature, difficulties have been reported in the analysis process of this high dimensionality data (Gromski et al., 2015) and there is still a need for methods and workflows that allow obtaining reliable results. Furthermore, when using metabolomics for biomarker discovery, data treatment methodologies need to model the discriminatory relationship between a two state clinical outcome variable (such as healthy vs. diseased) and more explanatory variables (metabolites or features). The final objective is then to propose a short list of 1–10 biomarkers, suitable for clinical utilization, meaning that within the modeling process, the simplest combination of metabolites producing a suitably effective predictive outcome has to be found.

**Figure 1:**
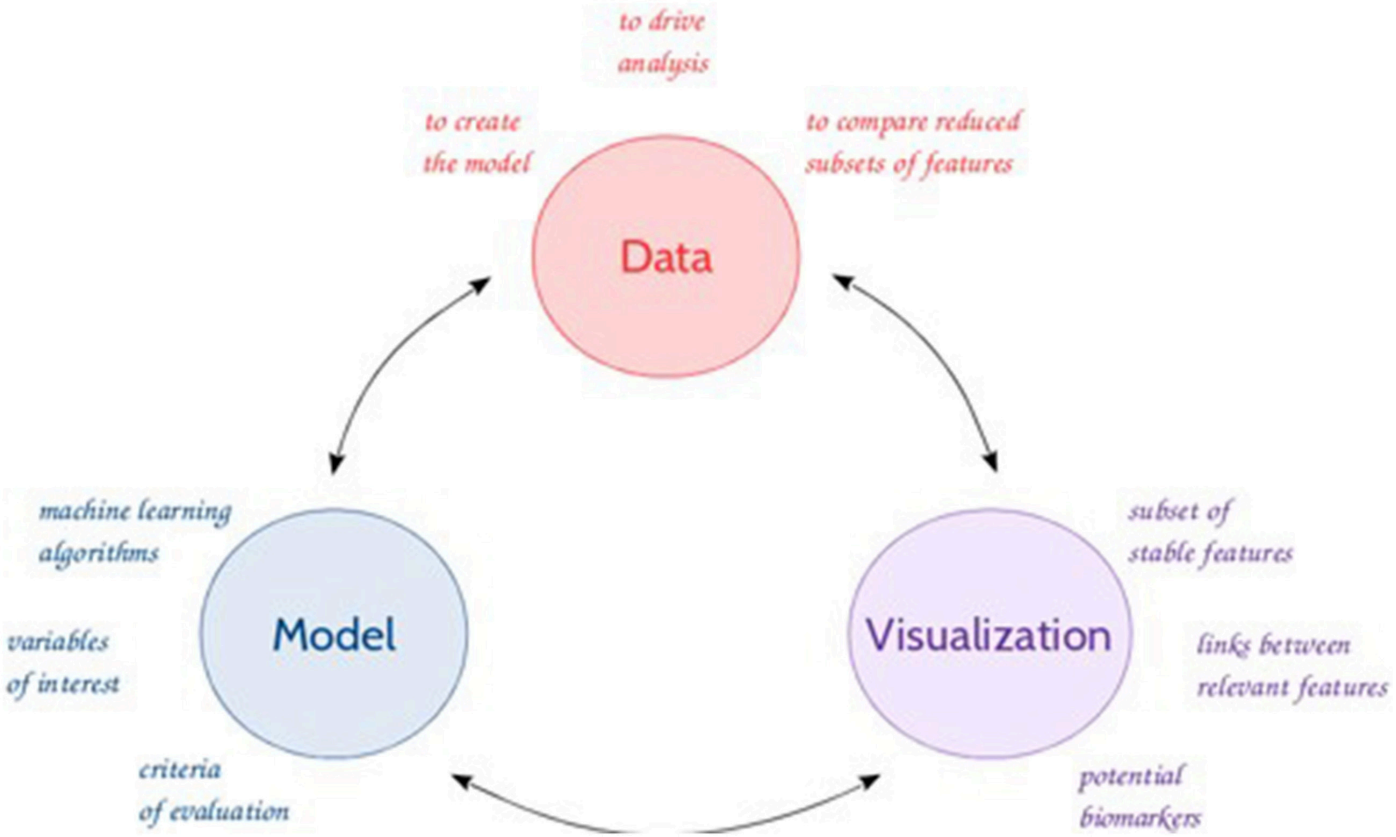
**General feature selection process**.

In this context, data mining tools, as well as machine learning algorithms are becoming of interest to analyze these large amounts of data and make accurate data-driven predictions. The use of (new) methods and tools enables the discovery of unknown patterns (or features) or relationships which can bring useful results and hypotheses to the experts (Giudici and Figini, 2009) in an iterative process (Figure 1). The emergence over the recent years of new metabolomics-based applications (e.g., high resolution MS, chemometrics methods) raised many new challenges, demanding further theories and techniques handling high-dimensional and complex data. Feature selection represents an integral component to successful data mining (Liu and Motoda, 1998; Guyon and Elisseeff, 2003). Biomarker discovery (Drabovich et al., 2013) is one of the most fundamental and challenging issue in feature selection (Liu and Yu, 2005; Saeys et al., 2007; Baumgartner et al., 2011). This issue can be addressed with a combination of different approaches from machine learning, multi-dimensional methods, data visualization, to statistics, such as regression techniques, and clustering and classification to identify relevant features with a discriminative power. Nonetheless, the selection and eventually the sequence of the appropriate techniques can be problematic as evidenced by several comparative studies (Gromski et al., 2014, 2015; Saccenti et al., 2014).

Different robust supervised learning techniques are commonly used in the analysis of metabolomics data (Issaq et al., 2009; Boccard et al., 2010; Xi et al., 2014), like partial least squares-discriminant analysis (PLS-DA), Principal component-discriminant function analysis (PC-DFA), linear discriminant analysis (LDA), Random forest (RF), and Support vector machine (SVM), all based on recognized advantages with specific limitations for each of the methods. RF belongs to the family of ensemble methods (e.g., bagging, Breiman, 2001; boosting, Freund and Schapire, 1997), and more specifically of classification trees. Introduced in 2001 by Breiman (Breiman, 2001), this approach becomes more popular and shows high performance for classification and discrimination in several and various application domains (Fan et al., 2011; Patterson et al., 2011). The principle of RF is to recursively generate a large number of binary decision trees, each of them being built from a bagged sample set randomly among the data. In RF, each tree is independently constructed using a bootstrap sample of the training data. The left out data, named out of bag (OOB) data, is used to calibrate the performance of each tree. It is a feature selection method including features ranking, based on the measure of each feature importance in the overall result. Alternatively, SVM, originally proposed by Vapnik (1998), is a multivariate supervised machine learning technique suitable for both classification and regression problems. It becomes more popular because of its robustness and its kernel approach. SVM works by depicting samples as points in a high-dimensional space that allows separating distinct classes of samples into distinctive regions. An optimal separation is the major goal of SVM method, since it attempts to directly find the best dividing hyperplane that has the greatest distance to the nearest training data point of any class, and therefore the widest margin. The small fraction of the samples/points positioning on the boundaries of these margins are referred to “support vectors.”

SVM and RF algorithms have been applied in the field of biomarker discovery (Chen et al., 2013; Gromski et al., 2014). RF is a highly accurate classifier, performing robust-to-outlier models. Its main advantage, as reported in the literature (Ho, 1998; Liaw and Wiener, 2002; Biau, 2012; Hapfelmeier et al., 2014), includes principally its power to deal with over-fitting and missing data, as well as its capacity to handle large datasets without variable elimination in terms of feature selection (Menze et al., 2009). It was successfully applied as a biomarker selection tool for metabolomic data analysis in several studies (Chen et al., 2013; Scott et al., 2013; Gromski et al., 2015), especially due to its resilience to high dimensionality data, insensitivity to noise, and resistance to overfitting, etc. Nevertheless, it generates different results, contrary to SVM which delivers a unique solution. As RF, the SVM technique has also been adapted for features selection purposes (Hermes and Buhmann, 2000; Weston et al., 2001; Guyon et al., 2002), and widely applied in various biological fields in which the feature size far exceeded the available number of samples, such as in metabolomic analyses (Mao et al., 2007; Frickenschmidt et al., 2008; Gromski et al., 2014), because of its potential to extract relevant chemical and biological knowledge from complex data. The main advantage of SVM is its flexibility in the selection of the kernel function that enables the separation of different sample classes. Nevertheless, this technique suffers from certain limitations, essentially its sensitivity to over fit the model selection criterion, which may introduce bias in the classification result (Cawley and Talbot, 2010) and the lack of transparency (quantifiers) of the results making rather abstract the identification of important variables. This obstacle can be circumvented by the identification of relevant features *via* the use of a SVM classifier combined with a recursive feature elimination (RFE) approach, as introduced by Guyon et al. (2002). Moreover, SVM enables solving binary problems and is ideal for case-control studies (Cortes and Vapnik, 1995; Vapnik, 1998). Therefore, these alternative approaches may be useful tools for generating various models through data reduction and feature selection, as well as providing more accurate results. However, different studies showed that the choice of the appropriate algorithms and workflow is highly dependent of the dataset characteristics and the objective of the data mining process (Scott et al., 2013).

In the context of the identification of early predictive markers of clinical outcome, few years before occurrence, in relatively homogeneous populations considered as healthy at the time of analysis, untargeted metabolomics data driven approach required a specific data processing enabling the discovery of subtle effects among a large amount of data. The objective of the present study was then to compare different feature selection methods and evaluate their capacity to select relevant features for further use in predictive models. It consists therefore in studying a full workflow inspired from Figure 1, describing the general feature selection process, using knowledge discovery and data mining methodologies to propose advanced solutions for predictive biomarker discovery from untargeted metabolomic data. The strategy was based on evaluating a combination of numeric-symbolic approaches, two common techniques from machine learning, RF and SVM, compared with classical univariate analyses ANOVA (Cho et al., 2008). This evaluation was done on the basis of performances of the final predictive models. Different feature selection approaches were applied either on an original metabolomic dataset or on reduced subsets. Filter methods based on the correlation coefficient and mutual information were tested to eliminate redundant/dependent features Then, supervised learning methods as SVM, RF, and SVM-RFE were used to rank the variables and select the most discriminative and predictive ones. This feature selection approach was based on different accuracy measurements, the “Gini” and “kappa” scores, as well as the weight “W.” In addition, univariate statistical tests were performed. A comparative study of the best k features obtained from the combination of these different approaches (10 methods in total) identified their degrees stability (1–10). A binary matrix of the form (N features × 10 data analysis techniques) based on the method of presence/absence of features was built. This matrix was the starting point for the application of the Formal Concept Analysis (FCA) method and the construction of concept lattices. According to the structure of the lattice, a set of features shared by complementary techniques were retained for the prediction step. A final post-processing phase focused on prediction, validation, and visualization and interpretation of the results, with the generation of correlation-based networks and association rules as complementary explicative data.

## Materials and methods

**Figure 2:**
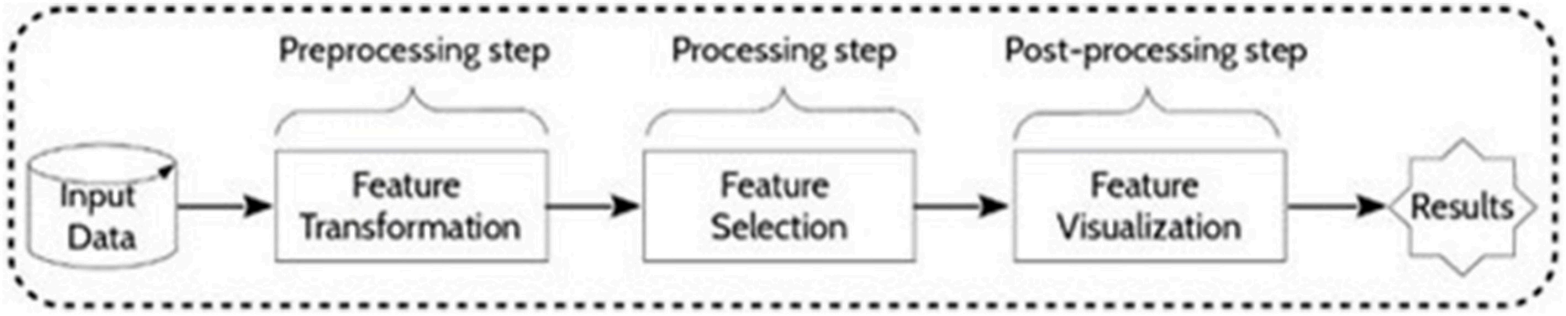
**General framework**. Main phases of metabolomic data treatment.

**Figure 3:**
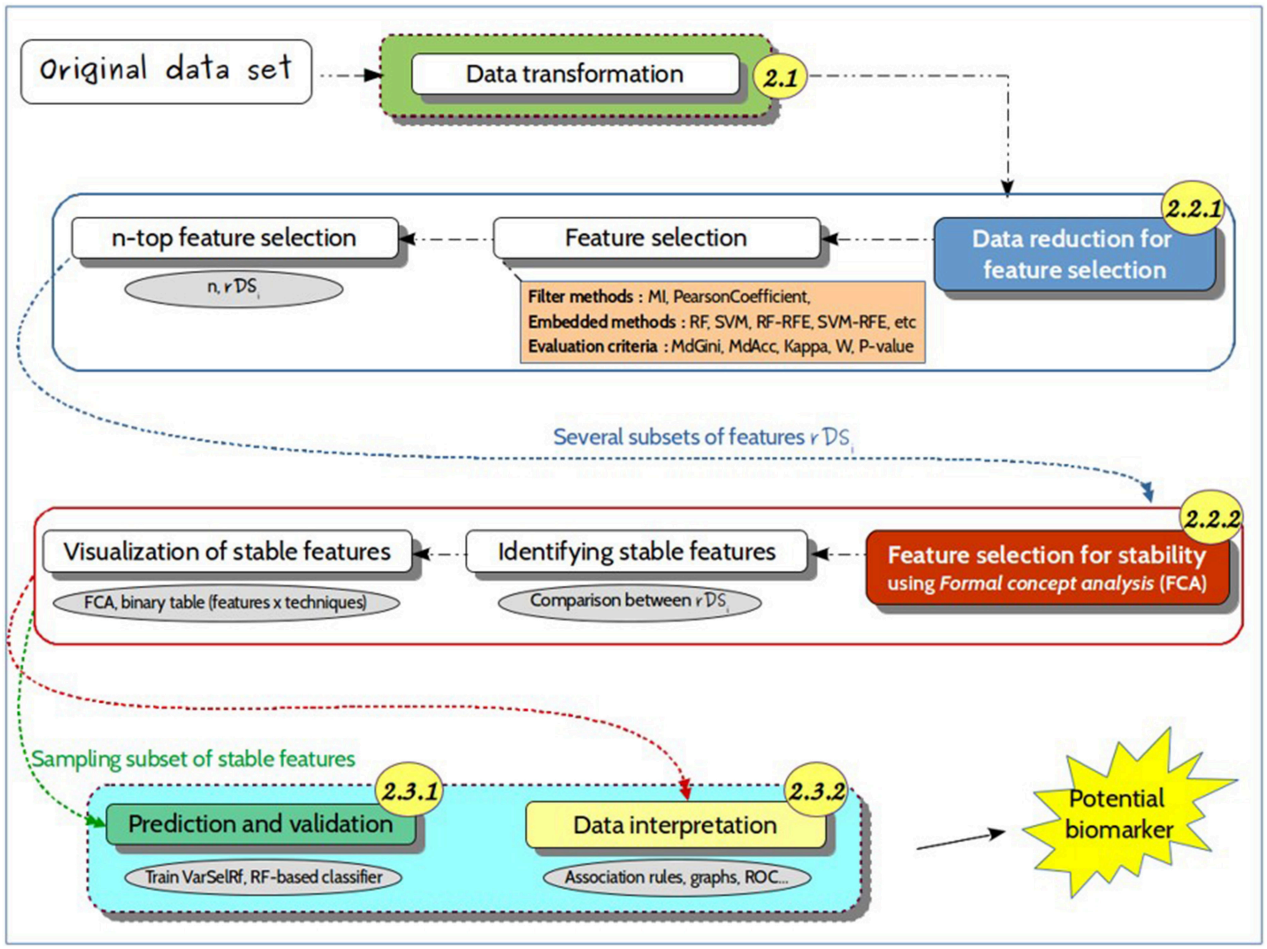
**Detailed approach**. Representation of the different steps of the proposed approach, from the untargeted metabolomics original dataset to the identification of predictive biomarkers. It includes (i) data transformation, i.e., noise filtering, scaling, to generate suitable datasets for feature selection methods; (ii) data reduction for feature selection, which consists in identifying relevant features for further use in predictive models; (iii) a prediction and validation step for discovering the best predictive markers. The numbers in the circles refer to the different sections of the manuscript for a more detailed description.

The present approach for feature selection from metabolomic data analysis comprises pre-processing, processing and post-processing steps (Figure 2). In phase 1, a transformation of the input collected data was performed by scaling the data. In phase 2, feature selection was conducted by some given machine learning algorithms. Then, a resulting reduced subset was used as input data for prediction. The performances of the final models were determined to finally evaluate the capacity of the alternative selection methods to identify the most relevant features. Figure 3 summarizes the process of the current approach with the three main phases and five steps.

### Data collection and pre-processing

Biological samples were obtained from a case-control study within the GAZEL French population-based cohort (n~20,000) (www.gazel.inserm.fr) (Goldberg et al., 2015). The GAZEL cohort received approvals from the National Commission for Data Processing and Freedoms (CNIL), the National Medical Council and the National Consultative Committee of Ethics, and the INSERM IRB. The volunteers gave written and informed consent for this study.

At risk male subjects (*n* = 111, 54–64 years old) with high body mass index (BMI, 25 ≤ BMI < 30), free of metabolic syndrome (MetS) at baseline, were selected. Cases who developed T2D at the follow-up (5 years later) were compared with Controls (matched for BMI and age). Baseline serum samples were analyzed using mass spectrometry-based untargeted metabolomics. Metabolic profiles of deproteinized sera samples were determined using an UPLC/QToF mass spectrometer (Bruker Impact HD2), equipped with an ESI source. Separations were carried out using an Acquity UPLC HSS T3 column (Waters) at a flow rate of 0.4 mL/min (Pereira et al., 2010). Data were acquired in positive ion mode with a scan range from 50 to 1000 mass-to-charge ratio (m/z). Samples were randomized within the analytical sequence using a Williams Latin Squares defined according to the main factors of the study. Data were processed under the Galaxy web-based platform (Worflow4metabolomics, Giacomoni et al., 2015), using first XCMS (Tautenhahn et al., 2008), followed by quality checks and signal drift correction according to the algorithm described by van der Kloet et al. (2009), to yield a data matrix containing retention times, masses and peak intensities that have only been corrected for batch effects, without any other normalization. This step included noise filtering, automatic peak detection and chromatographic alignment allowing the appropriate comparison of multiple samples by further processing methods.

### Feature selection methods

#### Data reduction for feature selection

This section presents the feature selection process, with a full description of each phase. All data analyses were performed using the Rstudio software (Version 0.98.1103 with R. 3.2.2) environment. This language offers a selection of packages suitable for different types of data and is available as a free software in the public domain.

Several feature selection methods are generally used in bioinformatics when the number of variables is large and the sample size is relatively small. Two different alternative type of feature selection methods can be used to reduce the dimensionality of data and identify variables with the best discriminant ability and predictive power: (i) embedded methods, which use a predictive model to give a score to the feature subsets, or (ii) filter methods, which use a proxy method (MutualInformation, PearsonCoefficient, Inter/intra class distance) to give a score to the feature subsets. Feature selection algorithms are divided into two categories:

1. Feature ranking: ranks the features by a metric and eliminates all features that do not achieve an adequate score;
2. Subset selection: searches a subset of possible features from the original set.

In the present study, reducing the dimensionality of the data is one of the most challenging step, requiring a careful choice of appropriate feature selection techniques. Two main type of methods were selected:

∘ The “filter” methods: consist in selecting variables using correlation coefficients and dependencies. As mentioned before, metabolomic data contain highly correlated features, which can be a drawback in some ranking methods such as some RF variable importance calculations (Gromski et al., 2015). To overcome this problem, variables were selected with a method using a coefficient of correlation (Cor) and mutual information (MI) criterion. Very highly correlated features were discarded to keep a reasonable number of variables to work with.
∘ The “embedded” methods (Lal et al., 2006): consist in searching for the optimal subset of features according to their usefulness to a given predictor. They consist in computing feature class based on supervised classifiers, as SVM and RF. These methods are guided by the learning process that offers a chance to build more accurate classifiers, and are based on a limited number of features to identify the most “relevant” ones.

In the context of feature selection, each feature has a score of importance that enables the identification of “relevant” features. For RF feature importance, two straightforward methods are provided: (i) *mean decrease in accuracy (MdAcc)*, which measures the importance of each variable to the classification; (ii) *mean decrease in Gini* index *(MdGini)*, which provides a measure of the internal structure of the data (Boulesteix et al., 2012). The general idea is to permute the values of each variable and measure the decrease in the accuracy of the model.

For SVM-RFE, which is a multivariate supervised approach based on a forward and backward sequential search, several metrics of feature importance are used, as the SVM weights “W” (i.e., the weight magnitude of features), the accuracy (Acc) and the Cohens Kappa (Kappa) metrics. The Kappa is a statistical measure comparing an observed accuracy with an expected accuracy (random chance).

**Figure 4:**
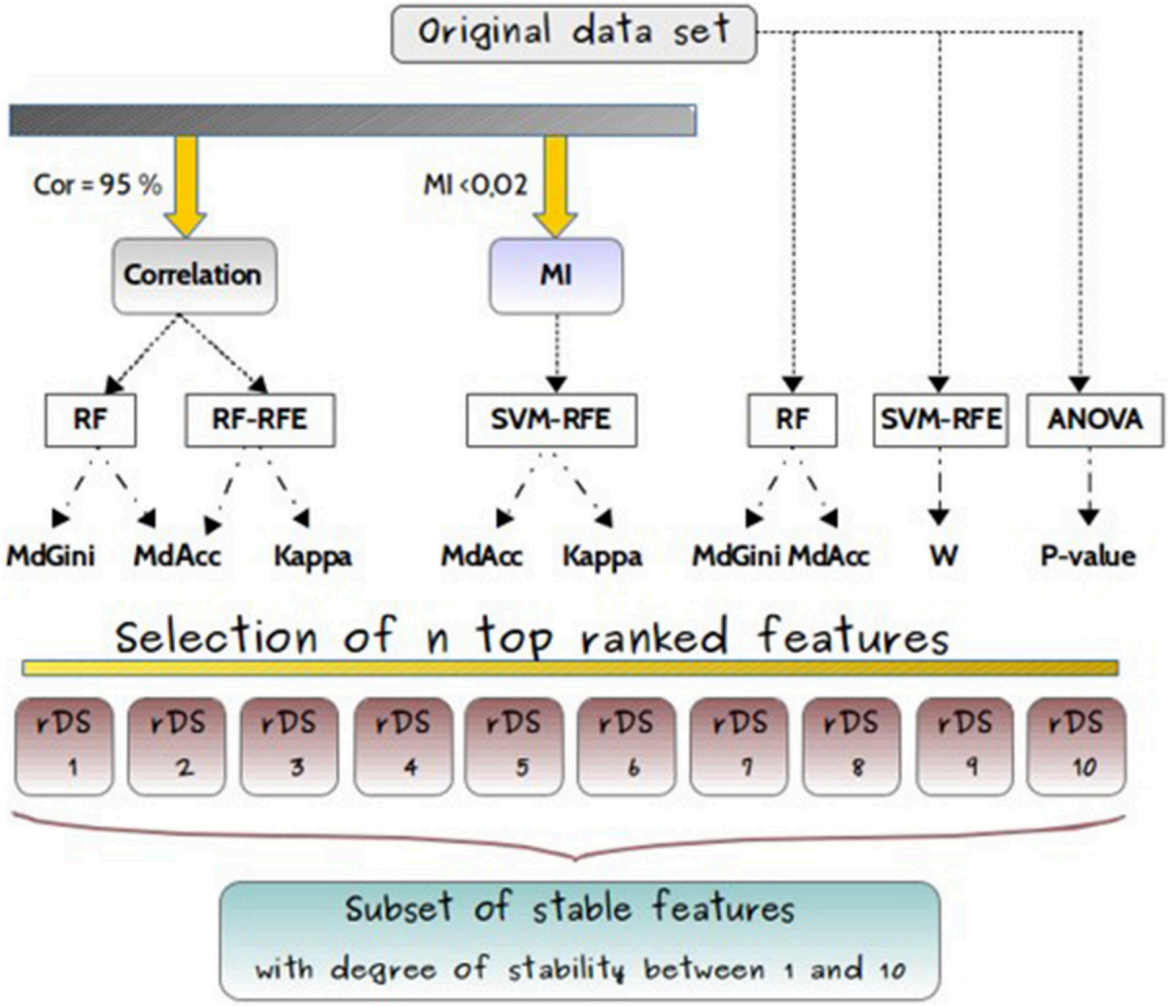
**Experimental design**. Experimental design for comparisons of feature selection methods (RF, RF-RFE, SVM-RFE, ANOVA), applied either on original dataset or after filters, based either on correlation coefficient (Cor) or on mutual information (MI), and used with different classifiers (MdGini, MdAcc, Kappa, W, *p*-value). It resulted in 10 different subsets, with different feature rankings.

The feature selection methods were either applied on the original dataset without filter, or with previous filters as shown in Figure 4.

##### Feature selection with no filter

The analysis was restricted to machine learning techniques (SVM-RFE or RF) that were applied directly on the original dataset to identify the optimal feature subsets. Firstly, the SVM-RFE with linear kernel as a classification method was chosen.

The weight values “W” of the decision hyperplane given by the SVM were generated, and sorted in a decreasing order to obtain the core set of features with the highest discriminative power. Secondly, the RF technique was performed on the whole dataset with the parameters described below for sub-dataset. The variables of the model leading to the smallest misclassification error value ranked according to the mean decrease accuracy method, were selected. In addition to these machine learning algorithms, an univariate statistical tool ANOVA was also applied since it provides stable results, by calculating the “*p*-value” of each feature in the objective of identifying the significant ones. Verification of ANOVA assumption was performed prior to analyses (see Supplemental Data). The choice of ANOVA was justified by its common use in metabolomics, in particular due to the possibility to add co-variables if necessary. An ascending order of the 1195 variables according to their *p*-values was performed. Consequently, three additional subsets of features “SVM-RFE-W,” “RF-Acc,” and “*p*-value” were obtained with these different variable rankings.

##### Feature selection based on correlations

Two approaches namely “Cor-RF” and “Cor-RF-RFE” were applied. They first removed redundant features using a correlation coefficient criterion, and then used RF or RF-RFE methods to select the “n” best ones based on MdGini, MdAcc, Acc and Kappa scores. Concerning the correlation filter, the selection was conducted using the “findCorrelation” function from the “Caret” R package. Features which were highly correlated were filtered out: one feature was kept out of each set of correlated ones. In order to limit the loss of information, we used Pearson correlations with a threshold set to 0.95. Consequently, a reduced subset was generated and used as input for analysis with RF. To train RF on this new subset, the “randomForest” R package (Liaw and Wiener, 2002) was used, with the following standard parameters: number of trees = 2000 and number of variables selected at each node = 2 × √ number of features in the subset. Nonetheless, as correlation values between variables were still high, we furthermore adapted the RFE approach with RF, using the Caret R package. The feature selection process for the four subsets was replicated *t* = 50 times, and the model leading to the smallest “OOB error” was selected.

##### Feature selection based on MI criterion

An alternative method “MI-SVM-RFE” was tested. It first filtered out the dependent variables by means of mutual information criterion (MI), and then selected the “n” best feature subset based on SVM-RFE feature selection with calculation of accuracy and kappa scores. The “mRMR.data” and “mim” functions of the “MRMR” R package were used to compute the mutual information matrix over the full data set. The average mutual information of variables was then calculated and a MI threshold of 0.02 was set. All the features whose MI values were smaller than the threshold were selected, since it is recognized that high values of MI are indicating a large uncertainty needing to be reduced (Wang et al., 2013). An independent feature subset was obtained, and used as input data for applying the SVM-RFE technique within the “kernlab” R package and the “rfe” function with SVM model. This method enabled to obtain variables using accuracy and kappa metrics. As a result, two new subsets “MI-SVM-RFE-Acc” and “MI-SVM-RFE-Kap” were generated.

Cross-validation *via* leave-one-out CV (LOOCV) was performed for evaluating the best models (Kohavi, 1995) after having compared various matrix of performance (see Supplementary Data).

##### n-top feature selection

The subsets of “n” highly ranked features derived from each approach within different metrics of importance (MdGini, MdAcc, Kappa, W, and *p*-value), were selected as the results of the first dimensionality reduction of the original data set. Therefore, multiple subsets of reduced data rDSi where i ∈ {1…n} containing the top ranked were obtained as output and exported for further analysis of their stability.

#### Stable features selection

This step focused on comparing all the reduced subsets (rDSi) of obtained features. For this propose, a presence-absence table of features × data-analysis-techniques was conducted (see Supplementary Table), where the objects (rows) are the features and the variables (columns) are the data analysis techniques (10 techniques). Each feature had a degree of stability identified from the obtained binary table, where the most stable features are those existing in all the reduced subsets.

#### Visualization of stable features

FCA is an unsupervised technique based on a mathematical theory of data analysis. Introduced by Ganter and Wille (1999), it enables deriving a concept hierarchy by means of formal contexts, data tables that represent binary relations between objects and their attributes. In this theory, each concept in the hierarchy represents a set of objects (the “extent”) and a set of attributes (the “intent”) such that the extent consists in all objects that share the given attributes, and the intent consists in all attributes shared by the given objects. Each sub-concept in the hierarchy contains a subset of the objects in the concepts above it. FCA was applied for a visualization purpose for viewing the different relationships between the variables from one hand, and between variables vs. applied techniques from the other. It was applied on the binary table previously obtained, and a concept lattice of “p” concepts was then generated (using the “ConExp” tool, Yevtushenko, 2000).

### Post-processing phase

The last phase was dedicated to the prediction, validation, and interpretation of data:

#### Prediction and validation

For prediction, the subset of stable top-ranked features was selected and different alternative techniques were used.

To perform prediction based on RF, the dataset was sampled into a first training set (on average 75% of the samples) to fit the model with the construction of trees and a validation set for estimation of the classification accuracy. Therefore, the RF model was firstly trained over the selected subset (retained from the concept lattice), and then the classifier was validated on the corresponding test sets using the usual mean classification error. Evaluation of the model was performed using cross validation. Randomly selected subspaces were considered and 100 replications of the selection procedure were performed.

Alternatively, a second variable selection algorithm using random forests was applied for prediction purpose: “VarSelRF” (Díaz-Uriarte and de Andrés, 2006). It is based both on a backwards variable elimination (for the selection of small sets of non-redundant variables) and on a selection with on the importance spectrum to provide a reduced set of predictive features. Since it is a subset feature selection algorithm that generates directly a reduced set of predictive features, it was directly applied on the 48 features subset without splitting the data. The “varSelRF” function proposed by “VarSelRF” R package was applied. Several replications (100 times) were carried out; for each replication, a different reduced set of relevant features was generated since the algorithm aimed at reducing the set of predictive variables until obtaining the lowest OOB error rate.

Finally, logistic regression was performed on the same reduced subset and compared with the same analysis performed on a dataset containing significant features (after ANOVA). In a first step, a partial logistic regression was performed separately for each feature. Only those whose coefficient had a *p*-value lower than 0.25 were selected. In a second step, paired-wise Pearson correlation coefficients were calculated. Then, in order to remove the information redundancy and collinearity between features, and to select only the 10 most potentially predictive ones, features were eliminated one by one, until obtaining a maximum correlation coefficient in the matrix of 0.5, the one of the two features the most correlated to others in the matrix being eliminated. At this step, the elimination of the remaining features was based on the lowest correlation with the outcome factor and the less significant *p*-value in partial logistic regression. Then, the 10 remaining features were introduced in a multiple logistic regression model, which was finally automatically reduced using the AIC values in a stepwise method (R package “stats”).

Six common evaluation metrics were employed (sensitivity, specificity, accuracy, precision, OOB error, and misclassification rate), defined in Supplementary Table 1. “Specificity” also called true negative rate, measuring the proportion of correctly identified negative instances relative to all real relative ones. It evaluates then the efficiency of the classifier in identifying the true positive features (“Sensitivity”). The “Precision” rates the predictive power of a method, by measuring the proportion of the true positive instances relative to all the predicted positive ones. The “Accuracy” or “G-score” evaluates the overall performance of the feature selection methods, since it measures the predictive model capacity to classify correctly both positive and negative instances. Additional assessment criteria can be calculated besides the evaluation of the optimal feature sets based on the above-mentioned metrics: the “misclassification rate” or “error rate,” is equivalent to “1—accuracy.” It refers to the misclassification rate of the learning model, by estimating the proportion of wrongly classified features (negative and positive equally). The size of the feature set has an impact on the performance of the final model.

Most machine learning methods need to resort to cross-validation for the estimation of a classification error, but RF can intrinsically estimate an OOB error in the process of constructing the forest.

#### Data interpretation

The results of predictive features and models previously obtained were considered for Receiver Operating Characteristic (ROC) curves constructions. ROC curve is a non-parametric analysis, which is considered to be one of the most objective and statistically valid method for biomarker performance evaluation (Xia et al., 2013). These analyses were performed using the ROCCET tool (http://www.roccet.ca) for the univariate and the multivariate RF-based analyses and the pROC R package for prediction based on logistic regression (Robin et al., 2011) with calculation of the area under the curve (AUC) and confidence intervals (CI), calculation of sensitivity, specificity. The *p*-values of the predictive variables were also computed using *t*-test, and the core set of best features with the smallest *p*-values and the highest accuracy values was selected to finally obtain a short list of potential biomarkers. To evidence the relationships existing between these predictive variables, a correlation network was built using a correlation matrix based on Pearson coefficient. Association rules (Agrawal et al., 1993) based on a series of operations (numeric attributes values discretization on 4 ranges, and association rule mining on the resulting data set) were also performed using the WEKA tool (Witten and Frank, 2000).

## Results

### Data characteristics

The dataset included measurements of 111 individuals divided in two groups: 55 belong to the class “1” (Case) and 56 to the class “−1” (Control). After noise filtration of metabolomic data, each subject was described by 1195 m/z variables, referred to attributes or features. Our objective was to reduce the dimensionality of this dataset and to extract the potential candidate biomarker discriminating cases from controls.

The metabolomic dataset showed 2.4% of the ions with correlation coefficient higher than 0.5 in the auto-correlation matrix, with 576 ions with a least one correlation higher than 0.8. Correlation networks of the ions with correlations higher than 0.5 showed highly correlated clusters due to both analytical and biological origins (Supplementary Figure 1). Moreover, 107 features were identified with a *p*-value lower than 0.1 after ANOVA, which represent around 9% of the original dataset. Fifty two (4.3%) were found to be significant after Benjamini-Hochberg (BH) correction. This result confirmed the fact that the discovery of predictive markers of pathology development years before it appears, implies the identification of subtle effects, reinforcing the need of adequate data mining methods.

### Data pre-processing

Data transformation: Because a wide concentration range exists among the different detected metabolite, a data pre-processing is necessary to adjust the importance allocated to the variables in some of the fitting models chosen. Thus, before applying SVM, data were transformed, using a Unit-Variance scaling method, which divides each variable intensity by its standard deviation; so that all variables have the same chance to contribute to the model as they have an equal unit variance.

### Feature selection and stability

Because of the large number of features derived from metabolomic data inducing intensive computation, it is crucial to reduce the feature dimension. In this study, the aim was to find a subset of the original dataset enabling the identification of relevant features and the decrease of the overfitting risk. The choice and use of appropriate features selection algorithms are of great importance to be able to build good models.

After feature selection based on correlation criterion, a subset of 963 features was generated and used as input for RF analysis. Four different subsets of the 963 ranked features “Cor-RF-Acc,” “Cor-RF-Gini,” “Cor-RF-RFE-Acc,” and “Cor-RF-RFE-Kap” were generated. Only 590 independent features were obtained using the Mutual Information technique resulting in two SVM-RFE subsets, named “MI-SVM-RFE-Acc” and “MI-SVM-RFE-Kap.” In order to compare the different subsets, the first 200 ranked features from each subsets were finally retained. When no filter was applied, the first 178 ranked common features were directly selected from the original dataset, except for ANOVA where only 107 features with *p*-value lower than 0.1 were filtered. Then, to analyze the relative importance of individual features and to enable a comprehensive interpretation of the results, the reduced subsets of features rDSi were combined for comparison. This feature-technique binary table was used to emphasize the most stable features presented in the Supplementary Table 2 (see Supplementary Data), on which the FCA technique was applied. Two hundreds and seventy six concepts were obtained from the derived concept lattice.

**Figure 5:**
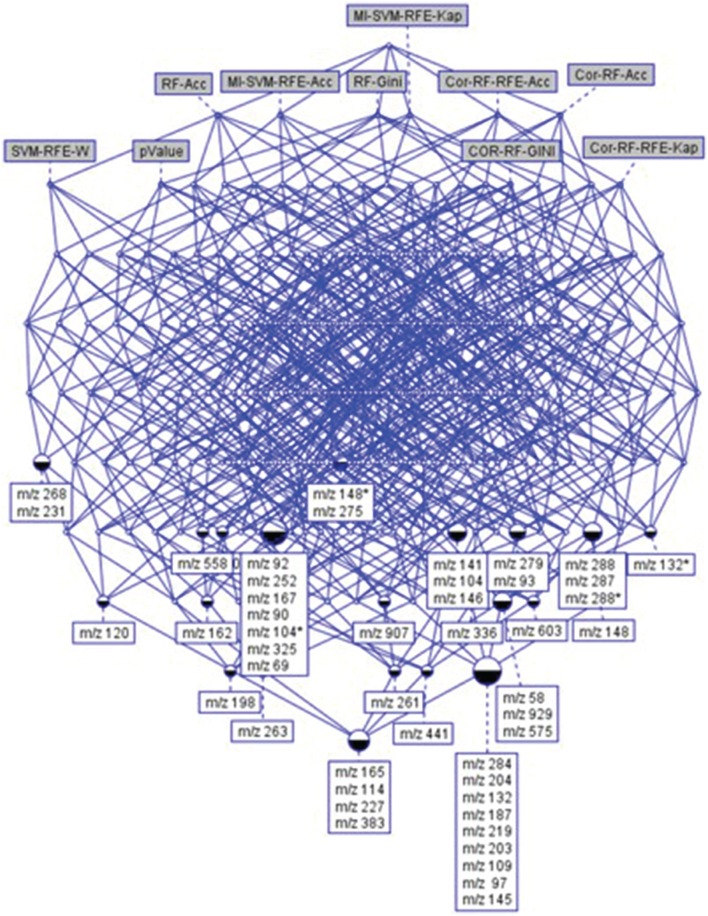
**The concept hierarchy derived from 48 × 10 binary table of Supplementary Table 1**. It highlights the relationships existing between top-ranked features and selection methods. It allows visualizing the common features (ions) selected by a set of methods.

In this study, we were looking for the most stable features according to the different data analysis techniques. The feature selection process is not a simple task in biomarker discovery, as it requires optimization of the biomarker usefulness regarding the biological relevance, but also the number of metabolites used in the predictive models. A subset of features common to at least 6 techniques was selected. This choice allowed obtaining results from complementary methods, ensuring the selection of some relevant features that could have been removed by filters, while keeping a reasonable dataset size. It resulted in the identification of 48 features (Figure 5). In the present study, as logistic regressions were used, this number of selected features looks adequate for being introduced in the process of predictive model construction. Among the 48 features, 39 were significant after ANOVA (with BH correction). Similar results were obtained with a non-parametric test in comparison with ANOVA (see Supplementary Data).

### Prediction

To meet the final objective of usefulness of the discovered markers in clinical practice, two main parameters need to be optimized: the biomarker performance and the number of metabolites used in the predictive models.

#### Using random forest

The impact of each feature on the model accuracy is essential in a biomarker discovery approach. Thus, the RF classifier was trained on the subset containing the 48 features based on LOOCV and using the MdAcc method to rank the predictive variables. A confusion matrix was generated enabling the evaluation of the model performance. A new subset “48-Rf-acc” of the ranked features was generated. From this, 5 additional subsets “40-Rf-acc,” “30-Rf-acc,” “20-Rf-acc,” “10-Rf-acc,” and “5-Rf-acc” were obtained, containing respectively 40, 30, 20, 10, and 5 highly ranked features.

Table 1 summarizes the obtained scores from the six common evaluation metrics, starting from the subset of 1195 of the whole dataset, to the one of 200 variables (including the 200 best ranked features according to RF with MdAcc from the original data set) and to the most reduced one of 5 variables (obtained from the selection of the 5 best ranked features according to RF MdAcc on the subset of 48 stable features). The performance of biomarker selection methods can be assessed by these evaluation criteria which measure the ability of the aforementioned learning algorithms to select a feature set that allows a good prediction regarding the group of interest. They are calculated from the confusion matrix constructed from the distribution of true positives (right prediction as positive), false positives (predicted positive but in reality negative), true negatives (right prediction as negative), and false negatives (predicted negative but in reality positive).

**Table 1:** **Performances of the models trained by RF**.

**Metrics**	**Sensitivity**	**Specificity**	**Accuracy**	**Precision**	**Misclassification (%)**	**OOB error**
1195-Rf-acc	0.81	0.65	0.73	0.71	27	0.261
200-Rf-acc	0.86	0.82	0.84	0.84	16	0.154
48-Rf-acc	0.93	0.80	0.87	0.83	13	0.131
40-Rf-acc	0.85	0.88	0.87	0.87	13	0.131
30-Rf-acc	0.83	0.90	0.87	0.90	13	0.131
20-Rf-acc	0.90	0.85	0.88	0.86	12	0.119
10-Rf-acc	0.85	0.86	0.85	0.85	15	0.142
5-Rf-acc	0.86	0.85	0.85	0.86	14	0.142

*Table of performance of 9 models trained by RF on different datasets. Six common evaluation metrics were used*.

The results showed that training RF from the whole data set provided the lowest performance model. However, by reducing the data dimensionality to 48 features allowed obtaining better values for most of the metrics. No subset outperformed all the others. Thus, the smallest subset of the 5 top ranked stable variables (“m/z 219,” “m/z 268,” “m/z 145,” “m/z 97,” and “m/z 325”), enabling a good accuracy classification of case-control subjects, was retained.

#### Using VarSelRf

Since 48 features still represent a consequent number of variables, and all may not necessarily be crucial in the RF prediction, VarSelRf was applied from this subset to obtain a RF model on a reduced number of features. The stable variables were retained from the different replications. The results revealed 5 predictive variables common to all repeated tests: “m/z 145,” “m/z 162,” “m/z 263,” “m/z 268,” and “m/z 97.”

Two features were common with the results from the RF method: “m/z 268,” and “m/z 97.”

#### Using logistic regression

The variable selection from logistic regressions performed on the 48 feature dataset resulted in 10 features, and 5 top variables were retained for the best model: “m/z 148,” “m/z 167,” “m/z 198,” “m/z 268,” and “m/z 288.”

The variable “m/z 268” was found to be the only common feature to all models. All final predictive models included 5 variables, partially different depending on the technique. Finally, a total of 11 predictive features were obtained using the three approaches. Table 2 presents the rank of these variables according to the feature selection methods. All the SVM-based feature selection methods did not achieve a good ranking. For the RF-based methods, RF-Gini gave the best results. However, the best ranking was obtained using ANOVA.

**Table 2:** **Ranking of the 11 best predictive features**.

**Features**	**RF-MdAcc**	**RF-MdGini**	**Cor-RF-MdGini**	**Cor-RF-MdAcc**	**Cor-RF-RFE-Acc**	**Cor-RF RFE-Kap**	**MI-SVM-RFE-Acc**	**MI-SVM-RFE-Kap**	**SVM-RFE-W**	**Anova-*p*-value**
m/z 145	1	1	1	2	46	53	100	125	323	2
m/z 97	2	2	3	1	142	185	63	67	159	3
m/z 325	5	5	7	5	210	220	38	37	1118	8
m/z 268	9	6	–	–	–	–	168	181	22	4
m/z 263	8	7	5	7	198	249	28	27	166	5
m/z 219	13	13	13	12	84	76	61	65	1022	12
m/z 162	104	31	20	26	211	221	39	38	103	17
m/z 288	167	36	25	29	140	152	–	–	976	22
m/z 148	43	47	27	86	87	98	66	70	471	38
m/z 198	101	71	150	496	48	36	70	84	167	34
m/z 167	48	50	45	24	505	586	144	154	13	39

*Ranking of the 11 best features according to 10 feature selection methods*.

### Prediction evaluation

Table 3 shows the identified predictive metabolites ranked by the AUC of their univariate ROC curves. The data revealed that five features presented AUC values higher than 0.75 considered as fair values, and *t*-test lower than 10E-5.

**Table 3:** **Table of performance of the 11 best features**.

**Features**	**AUC**	***t*-tests**	**95% CI**
m/z 145	0.795	1.448E-6	0.657–0.896
m/z 97	0.787	1.597E-6	0.657–0.898
m/z 325	0.773	2.233E-5	0.627–0.896
m/z 268	0.759	4.564E-6	0.614–0.866
m/z 263	0.753	5.996E-6	0.642–0.874
m/z 219	0.712	1.177E-4	0.162–0.798
m/z 162	0.656	0.00195	0.225–0.710
m/z 288	0.634	0.00499	0.252–0.708
m/z 148	0.630	0.01778	0.238–0.624
m/z 198	0.619	0.01368	0.197–0.594
m/z 167	0.541	0.01796	0.190–0.715

*Ranking of the 11 best predictive features by the AUC value from univariate ROC curves*.

In multifactorial diseases as T2D, a combination of a multiple “weak” multivariate model instead of a single “strong” individual markers often provides the required high levels of discrimination and confidence. Therefore, the three multivariate model performances were evaluated and compared (Table 4) using ROCCET models (RF with inner parameters: 500 trees and one third of the features at each node). The results showed that the multivariate models are more accurate than the ones obtained with the single features, with AUC higher than 0.82. Prediction based on logistic regression from the 48 feature reduced dataset showed a better performance with a lower misclassification rate (18%) and the lowest false positive value. The same analysis performed on the ANOVA significant metabolites showed a much lower performance (27% misclassification).

**Table 4:** **Table of performance of the 5 predictive models**.

	**AUC**	**95% CI**	**Misclassification (%)**	**False positive**	**False negative**
RF	0.830	0.72–0.94	19.8	9	13
VarSelRF	0.845	0.76–0.94	22.5	14	11
Logistic regression	0.820	0.75–0.89	18.0	10	10
Univariate analyses—top 5	0.831	0.73–0.93	23.4	12	14
Univariate analyses—top 11	0.869	0.67–0.96	18.9	12	9

*Results from RF-based ROC curves*.

For comparison, we selected the five first features which have an AUC higher than 0.75, and a significant small *t*-test value for building a multivariate ROC curve. The combination of these single features did not show any improvement in prediction accuracy in comparison with multivariate models (Table 4).

**Figure 6:**
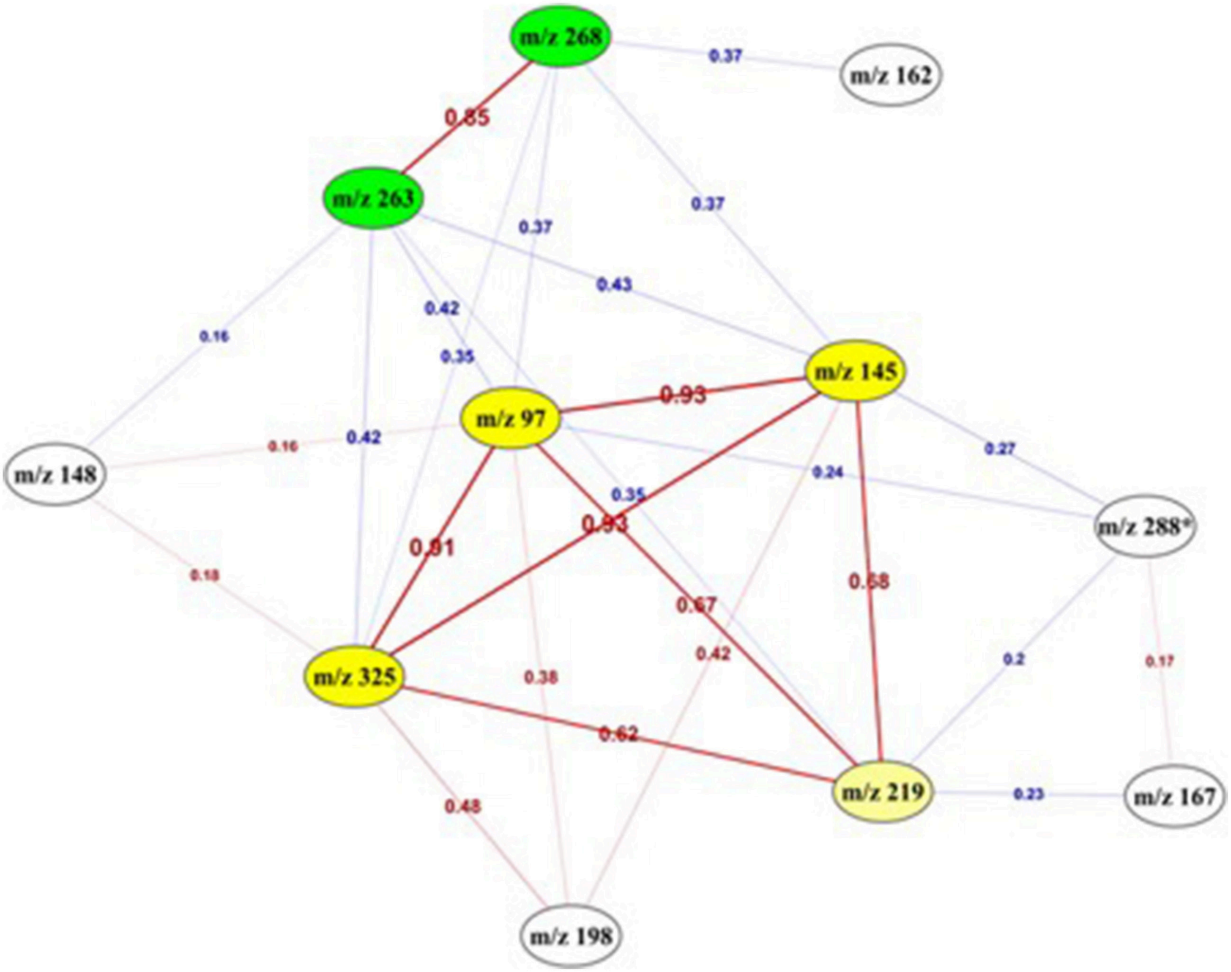
**Correlation network between the 11 top-predictive features**. Network built using Pearson correlation coefficient (indicated on the edges) between the best predictive features. Red edges: positive correlations, Blue edges: negative correlations. It highlighted two highly correlated sub-networks (yellow and green).

To go deeper in the understanding of the results, the degree of relationship between each pair of variables was assessed by computing the correlation coefficient between all possible pairs of the 11 features. Figure 6 presents the deduced correlation network. The results showed that the “m/z 145,” “m/z 97,” and “m/z 325” features were highly correlated with a correlation value higher than 0.9. Moreover, “m/z 268” and “m/z 263” were also positively correlated between each other with a correlation value equal to 0.85 and formed another group of features.

This link was also confirmed by the following association rules that highlighted strong relationships and implications existing between the values of the 5 most correlated features, in the form of a numerical interval for each of them. The association “m/z 325” = (−inf −0.183386] “m/z 97” = (−inf − (−0.369165)] → “m/z 145” = (−inf − 0.004732] was obtained with a confidence^1^ value equals to 0.97. The same ascertainment for “m/z 268” and “m/z 263” was found according to the following association rule “m/z 268” = (0.944719 − inf) → “m/z 263” = (0.667287 − inf), with a higher value of the confidence metric (0.9). The combination of these best features revealed another strong association rule (confidence = 1) with the following implication: “m/z 268” = (−1.17646 – −0.115871] “m/z 325” = (0.183386 − 2.305548] “m/z 145” = (0.004732 − 2.127673] → “m/z 263” = (−1.588695 – −0.460704].

## Discussion

### Feature selection

One of the objective of untargeted metabolomics in clinical studies is to provide a global view of biological processes involved in the development of pathologies. However, even if thousands of molecular features are quite useful for understanding involved pathways, they are not ideal for developing tests for clinical use. Therefore, performing feature selection is essential to discover new robust biomarkers being used for patient identification and/or stratification. In the present study, we assumed that a given biological phenomenon is not represented by all measured metabolites but simple sub-structures existing in the data that can be mathematically modeled.

In the context of identification of early/predictive biomarkers, our results showed that only a small fraction of detected features are discriminant, and they highlighted the importance of feature selection methods for obtaining the best performances of predictive models. In fact, despite RF is able to handle thousands of variables, when performed on the original dataset, it did not achieve a good accuracy in the prediction (27% misclassification). This result is in accordance with the observation made by Menze et al. (2009), with a stronger difference in the performance before and after feature selection. This could be related to the nature of the noisy data.

Univariate analyses showed a good ability to rank important features which is quite logical as this approach is considering each variable individually and therefore is non-sensitive to noise. *Post-hoc* corrections allowed avoiding false positive rate when multiple tests are performed. However, these approaches are remaining quite limited as they are not considering interactions between variables and only evaluate independent changes in metabolite levels. Untargeted metabolomics is a method of choice to detect thousands of metabolites reflecting the complexity of metabolism. In this context, multivariate methods are of great interest as they make use of all variables simultaneously and deal with the simultaneous relationship between variables, reflecting the orchestrated biological processes (Saccenti et al., 2014). However, these multivariate methods use correlations and co-variances between independent variables to predict the class belonging and they are sensitive to non-informative variables that affect the interpretation.

Our study illustrated the interest of RF-Gini as a complementary approach in addition to ANOVA for feature selection. These two techniques only could allow selecting the 48 best candidate features for prediction (nine of them were not selected with ANOVA alone). Moreover, the results showed that RF-Gini, as well as ANOVA, was one of the best method for feature ranking, especially for the best top-5 predictive features. Indeed, these top features were ranked among the 10 first ones with each techniques. This was also observed in previous published studies (Menze et al., 2009; Guo and Balasubramanian, 2012; Chen et al., 2013; Scott et al., 2013) with high dimension datasets from omics data. In particular, RF was shown to be robust to noise and outliers, and a technique of choice to avoid overfitting.

The choice of appropriate feature selection method is highly dependent of the dataset characteristics. And it is clear that so far, there is no universal classifier and feature selection is still not currently widely use (Scott et al., 2013; Gromski et al., 2014). Therefore, from these results, our recommendation would be to explore the combination of ANOVA and RF-Gini methods for reducing the dimensionality of such datasets, especially when predictive models are being built.

### Prediction

For clinical purposes, a short list of very limited number of robust biomarkers is necessary and therefore our objective was to end up with a predictive model showing good performances. In this area, classical approach is based on linear logistic regression, removing correlated features but needing a small number of variables. Using this technique on a reduced dataset enabled us to obtain the best performances in terms of accuracy and number of false positive. In contrary, RF can handle lots of variables but we realized that the performances of the model were better on a reduced subset of features. At the same time, as modeling complex pathological processes requires the use of large enough set of features/variables for prediction, models using too restrictive datasets are also not the most powerful ones (e.g., RF-based ROC curve models with 11 vs. 5 features). Moreover, a close examination of the relationships between the best predictive features could contribute to a better understanding of the results. In fact, within the 11 best features, quite strong correlations and associations were found. These additional data could contribute firstly, in structural elucidation of metabolites (one parent ion should be strongly correlated to its in-source fragments), and secondly in the biological interpretation as metabolites from a same involved metabolic pathway should be linked. This will allow having a vision of patterns of changes, complementary to the contribution of each identified metabolites in prediction of the clinical outcome.

In the present experimental conditions and using the same number of features (Gromski et al., 2015), univariate and multivariate modeling gave similar predictive results. However, in this study, we worked with standard/default RF parameters that could gain being optimized for better predictive performances.

When comparing predictive models obtained from logistic regression and RF-based methods, similar levels of accuracy and sensitivity were obtained. This observation is in concordance with the systematic review of Barber et al. (2014) on risk assessment tools for identification of prediabetes. The choice of a specific prediction tool can be motivated by the need of integrating other type of variables. RF could be recommended for high dimensional datasets from multi-omics, whereas logistic regression is more appropriate for clinical datasets in the objective of clinical use.

## Conclusion

Better understanding of complex biological mechanisms involved in pathological processes requires global approaches based on powerful analytical techniques as well as appropriate data mining methods able to handle large and complex datasets. The objective of this study was to explore alternative algorithms for feature selection from untargeted metabolomics data and evaluate the capacity of these methods to select the relevant ones for further use in prediction models. To fulfill this objective, one of the main issue is the optimization of two main parameters namely the biomarker performance and the number of metabolites used in the predictive model. Our results showed the interest of feature selection methods to identify hidden information in such high dimensional datasets. We have to keep in mind the challenge of biomarker selection with the identification of which variables (out of the many detected) are related to the observed discrimination between phenotypes. Due to the nature of metabolomic data (highly correlated and noisy), the results highlighted the importance of working on reduced datasets to obtain better performances in predictive models. Indeed, a combination of univariate and multivariate methods remains the best approach, as it allows combining the strengths of both techniques. In this study, RF-Gini combined with ANOVA provided the best feature selection for predictive biomarker discovery that will allow patient stratification few years before disease development. Our recommendation would then be to explore such techniques to process untargeted metabolomic data and reveal subtle metabolic changes. Even if they are still not usually applied, these data mining methods are essential tools to deal with massive datasets and contribute to elucidate complex phenomena. With this help, the experts of the scientific field will go deeper in interpretation, attesting the success of the knowledge discovery process.

## Author contributions

DG: performed design of the work, acquisition using feature selection methods and evaluation of the workflow; participated to the article writing; approved the final version; agreed to be accountable for all aspects of the work. MP: performed metabolomic data extraction, filtering and supervised statistical analyses; participated to the article writing; approved the final version; agreed to be accountable for all aspects of the work. MB: performed prediction using logistic regressions; approved the final version; agreed to be accountable for all aspects of the work. AN: conception and supervision of the work on feature selection methods and FCA analysis; participated to the article writing; revisited it critically for important intellectual content; approved the final version; agreed to be accountable for all aspects of the work. BC: performed data interpretation and integration; participated to the article writing; revisited it critically for important intellectual content; approved the final version; agreed to be accountable for all aspects of the work. EP: supervised metabolomic analyses and data treatments, conception of the workflow evaluation and the work on prediction; participated to the article writing; revisited it critically for important intellectual content; approved the final version; agreed to be accountable for all aspects of the work.

## Funding

Research funded by the Institut National de la Recherche Agronomique (DID'IT metaprogramme).

### Conflict of interest statement

The authors declare that the research was conducted in the absence of any commercial or financial relationships that could be construed as a potential conflict of interest.

## Footnotes

^1^The rule X → Y holds with confidence c if c% of the transactions in D (the set of all transactions) that contain X also contain Y.
