# Supplementary material for: Feature Selection Methods for Early Predictive Biomarker Discovery Using Untargeted Metabolomic Data
Source: Front Mol Biosci. 2016 Jul 8;3:30. doi: 10.3389/fmolb.2016.00030 (PMC4937038; doi:10.3389/fmolb.2016.00030)
Supplement: Supplementary file 1 [file DataSheet1.PDF]

Supplementary Material

Feature selection methods for early predictive biomarker discovery using untargeted metabolomic data

Dhouha Grissa[1], Mélanie Pétéra[2], Marion Brandolini[2], Amedeo Napoli[3], Blandine Comte[1] and Estelle Pujos-Guillot[1,2,*]

[1]INRA, UMR1019, UNH-MAPPING, F-63000 Clermont-Ferrand, France

[2]INRA, UMR1019, Plateforme d'Exploration du Métabolisme, F-63000, Clermont-Ferrand, France

[3]LORIA, B.P. 239, F-54506 Vandoeuvre-lès-Nancy, France

***Correspondence:**
Estelle Pujos-Guillot
estelle.pujos@clermont.inra.fr

1 Supplementary data

1.1 ANOVA assumption verification and comparison with non-parametric test

In this study, although the subject number (effective higher than 50 in each group) was high enough to assume normality, the variable distribution of metabolomics data was verified prior to data treatment. Around half of the variables showed a validated normal distribution, and none of them were found to have a particular non-normal distribution. Variances in the two groups of subjects were also compared, and were not found significantly different for most of the variables.

In a final step, results of ANOVA were compared with those of Mann-Whitney-Wilcoxon tests. Wilcoxon tests were less stringent than ANOVA. The best prediction models were obtained from around 50 variables. Consequently, with Wilcoxon tests, we would have selected variables which were significant in 7 of the 10 methods (stability of 7) instead of 6. As the best p-values of the two statistical tests (ANOVA and Wilcoxon test) were obtained with the same variables, the 48 features used for prediction models would have been nearly the same (the first features are ranked similarly).

1.2 Data resampling methods

In order to select the most appropriate method regarding the nature of our dataset, different resampling methods were tested. Various resampling techniques are used with different objectives: (i) to estimate the precision/prediction error of the model through the use of a data subset (e.g. Jackknife) or from a set of data points randomly replaced (e.g. bootstrap); (ii) to validate models by using random subsets (bootstrap, cross validation).

For example, several types of cross-validation can be applied on different kinds of models, including classification and predictive models to estimate their accuracy:

- leave-one-out cross-validation (LOOCV): leaves out every time a single sample ('n – 1' samples for the training set to fit a classification model and '1' for the testing set) × n where n is the sample size; this is similar to the jackknife;

- k-fold cross-validation (kFCV): randomly splits the data into k equal sized subsets; each is held out in turn as the validation set ('k – 1' for the training set and '1' for the validation) × k (with k = 5 or 10).

- Monte Carlo cross-validation (MCCV): randomly splits the data into two subsets by sampling, without replacement, some fraction of the data to form the training set, and the rest to the test set. This process is iterative, generating (randomly) new training and test partitions each time.

- random subsampling: randomly splits the data into training and validation sets (e.g., 75% of the data is used as the training set to build a classification model, and the other subset (e.g., 25% of the data) is used as the test set.

1.2 Results of data resampling method evaluation

Four resampling methods (10-Fold-CV, LOOCV, Bootstrap and MCCV) were assessed on models validation generated by RF and SVM on the original data set, using five common evaluation metrics (AUC, sensitivity, specificity, accuracy and precision). Supplemental Figure 2 revealed that generally LOOCV method showed better performance than the resampling tested approaches, since it had the highest values of evaluation measures, except for sensitivity and AUC which unveiled less good values than RF. Therefore, we retained LOOCV as a resampling method. On a general point of view, SVM was found with lower performances than RF for prediction. Therefore, the SVM algorithms were only used for feature selection.

2 Supplementary Figures and Tables

2.1 Supplementary Figures

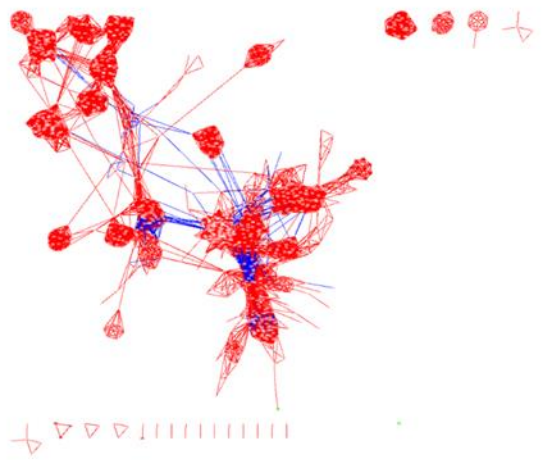

Supplementary Figure 1. Correlation network.

Correlation networks of the ions with Pearson coefficient correlations higher than 0.5. Positive correlations (red); negative correlations (blue).

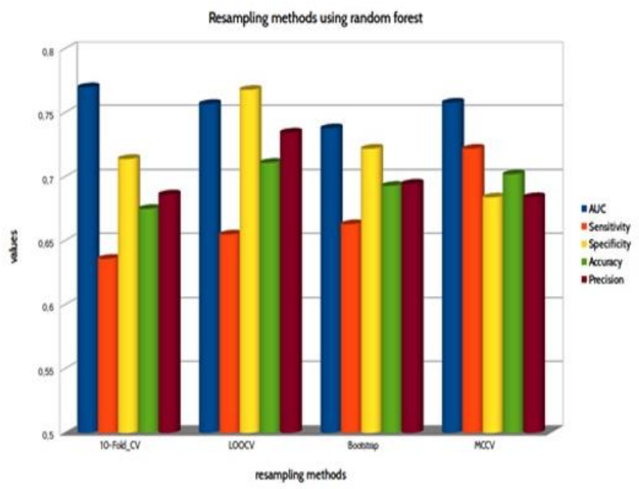

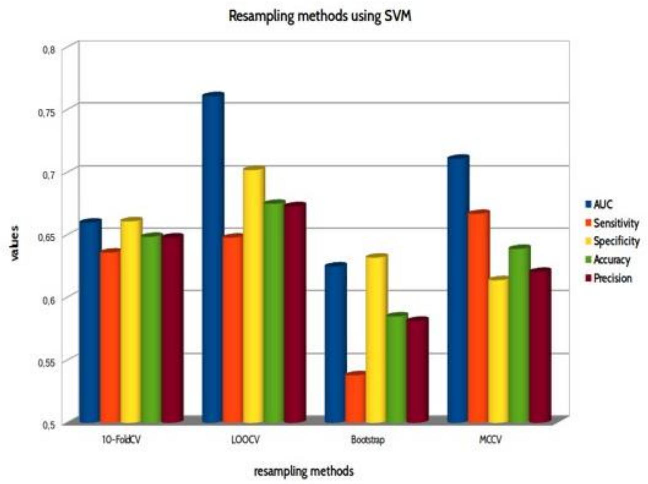

Supplementary Figure 2. Performance of resampling techniques.

Four resampling techniques were assessed on models generated by RF and SVM on the original dataset, using five common evaluation metrics.

2.2 Supplementary Tables

Supplementary Table 1. Evaluation criteria

Definition of six metrics to evaluate the performance of the models.

Measure	Equation
Specificity = True Negative Rate (TNR)	$\frac{tn}{tn+fp}$
Sensitivity = Recall = True Positive Rate (TPR)	$\frac{tp}{tp+fn}$
Precision	$\frac{tp}{tp+fp}$
F-measure	$\frac{(\beta^2+1)\times precision\times recall}{\beta^2\times precision+recall}$
Accuracy	$\frac{tp+tn}{tp+fp+tn+fn}$
Misclassification rate	$\frac{fp+fn}{tp+fp+tn+fn}$

Supplementary Table 2. Input binary table (features-techniques) describing the 48 selected stables features

Features / ions	COR-RF-Gini	Cor-RF-Acc	Cor-RF-RFE-Acc	Cor-RF-RFE-Kap	RF-Gini	RF-Acc	MI-SVM-RFE-Acc	MI-SVM-RFE-Kap	SVM-RFE-W	pValue
m/z 383	1	1	1	1	1	1	1	1	1	1
m/z 227	1	1	1	1	1	1	1	1	1	1
m/z 114	1	1	1	1	1	1	1	1	1	1
m/z 165	1	1	1	1	1	1	1	1	1	1
m/z 145	1	1	1	1	1	1	1	1		1
m/z 97	1	1	1	1	1	1	1	1		1
m/z 441	1	1	1	1	1	1	1		1	1
m/z 109	1	1	1	1	1	1	1	1		1
m/z 203	1	1	1	1	1	1	1	1		1
m/z 219	1	1	1	1	1	1	1	1		1
m/z 198	1		1	1	1	1	1	1	1	1
m/z 263	1	1	1		1	1	1	1	1	1
m/z 187	1	1	1	1	1	1	1	1		1
m/z 132	1	1	1	1	1	1	1	1		1
m/z 204	1	1	1	1	1	1	1	1		1
m/z 261	1	1	1	1	1	1	1	1	1	
m/z 162	1	1			1	1	1	1	1	1
m/z 284	1	1	1	1	1	1	1	1		1
m/z 603	1	1	1	1	1	1			1	1
m/z 148	1	1	1	1	1	1		1		1
m/z 575	1	1	1	1	1	1	1	1		
m/z 69	1	1			1	1	1	1		1
m/z 325	1	1			1	1	1	1		1
m/z 405	1	1			1	1	1		1	1
m/z 929	1	1	1	1	1	1	1	1		
m/z 58	1	1	1	1	1	1	1	1		
m/z 336	1	1	1	1	1	1	1			1
m/z 146	1		1	1	1		1	1		1
m/z 104	1		1	1	1		1	1		1
m/z 120			1	1	1	1	1	1	1	1
m/z 558	1	1			1	1	1	1	1	
m/z 231					1	1	1	1	1	1
m/z 132*	1	1	1	1	1		1	1		
m/z 93		1	1	1	1	1	1	1		
m/z 907		1	1	1		1	1	1	1	
m/z 279		1	1	1	1	1	1	1		
m/z 104*	1	1			1	1	1	1		1
m/z 90	1	1			1	1	1	1		1
m/z 268					1	1	1	1	1	1
m/z 288*	1	1	1	1	1	1				1
m/z 287	1	1	1	1	1	1				1
m/z 167	1	1			1	1	1	1		1
m/z 288	1	1	1	1	1	1				1
m/z 252	1	1			1	1	1	1		1
m/z 141	1		1	1	1		1	1		1
m/z 275			1	1	1		1	1		1
m/z 148*			1	1	1		1	1		1
m/z 92	1	1			1	1	1	1		1
